# Assessment of Intra-Oral Repair Systems for Veneered Zirconia and Zirconia Only

**DOI:** 10.3390/ma16041407

**Published:** 2023-02-07

**Authors:** Tugçe Merve Ordueri, Mehmet Muzaffer Ateş, Mutlu Özcan

**Affiliations:** 1 Department of Prosthodontics, Istanbul Medipol University, Istanbul 34083, Turkey; 2 Center for Dental Medicine, Division of Dental Biomaterials, Clinic for Reconstructive Dentistry, University of Zurich, 8032 Zurich, Switzerland

**Keywords:** bond strength, dental materials, direct porcelain repair, indirect porcelain repair, zirconia, prosthodontics

## Abstract

The aim of this study was to compare bond strength resin composites to porcelain laminate veneers in the indirect repair method to composite resins used in the direct repair method for cases of porcelain veneer fracture of zirconia-based fixed dental prostheses. In the study, the groups were formed with different percentages of areas to be repaired to mimic porcelain fractures in the mouth. The experimental group of veneered zirconia were as follows: Group A = 100% Zr surface; Group B = 70% Zr, 30% porcelain surface; Group C = 50% Zr, 50% porcelain surface; Group D = 30% Zr, 70% porcelain surface; Group E = 100% porcelain surface. The repairs of the specimens were made using composite resin systems in half of the groups and using porcelain laminate veneers in the other half. Specimens were embedded in acrylic blocks before surface treatments and repairs were applied. After surface conditioning, laminate veneers were applied to the first half of the groups, and composite repair systems were applied to the second half of the groups. After all specimens were aged by thermal cycling, their bond strength values were measured using a Universal Testing Machine, and the obtained data were recorded. The specimens were examined with a stereomicroscope and classified according to failure types (adhesive/cohesive/mixed). Bond strength values were evaluated based on independent-samples *t*-test statistics. According to the comparisons among the groups, the bond strength of the indirect repairs made with the laminate material was higher than the bond strength of the repairs made with the composite. There was a statistically significant difference in favor of the indirect repair groups among all groups except for Group C. The highest bond strength was found in Group A in the indirect repair method, while the lowest bond was found in the direct repair method in Group E. Adhesive failure was mostly seen in the groups that were repaired with the composite.

## 1. Introduction

One of the current aims of dentistry is to replace the missing tooth tissue and bring back the aesthetic, functional and biological balance of the patient’s teeth [1]. Fixed prosthetic restorations are often used to replace missing teeth. Metal-based ceramic restorations have been used as fixed prosthetic restorations since the mid-1950s [2]. The use of metal-based ceramic restorations in fixed prosthetitic applications is associated with the superior mechanical properties of metals [3]. However, known aesthetic and biological problems have increased the use of all-ceramic systems [4]. Today, all ceramic restorations are used more frequently as an alternative to metal-supported restoratins for reasons such as more succesful aesthetic results, high biocompatibility, and durability [5]. 

It is known that the most common complication in zirconia-based restorations is the delamination or chipping of the ceramic from the substructure [6]. This failure can be caused by many factors such as parafunctional habits, patient-related factors, lack of occlusal balance, material properties and fatigue, forces caused by premature contacts, insufficient substructure support, insufficient bond strength, and differences between the thermal expansion coefficients of the substructure and superstructure materials [7]. This failure of the superstructure porcelain, or on the connection surface, requires restoration, as it affects function and aesthetics, and this is an undesirable situation for the patient and the physician from a clinical point of view. While a restoration is being removed from the mouth, the abutment teeth may be damaged and cause patient dissatisfaction. Producing a new prosthesis will also lead to loss of time and additional costs [8]. If the broken restoration is a part of a fixed multi-member prosthetic restoration, especially if this restoration is located anteriorly and there is no other indication to remove the prosthesis, it creates a difficult situation for the patient and the physician. The problem becomes greater if the fractured porcelain is a part of a precision-retained or telescopic, removable partial denture, or a one-piece over-implant restoration [9].

Since it is not possible to add new porcelain in the mouth due to the structure of porcelain, intraoral porcelain repair methods have been developed. This way, pain that may occur during the removal of the restoration, damage to the remaining teeth, loss of time, and additional technician costs are prevented for the patient and the physician [10]. Repairing the broken restoration without removing it from the mouth is satisfactory for both the patient and the physician. After the repair process is performed, the connection between the repaired part of the restoration and the remaining part should be strong and enough to withstand functional forces and aesthetically pleasing [11]. Repair methods are divided into two groups as the direct and indirect methods [12]. The direct repair method is the most preferred method of applying the composite resin material directly to the broken surfaces in the prosthesis. The indirect method, on the other hand, is the method of removing the restoration intact and repairing it in the laboratory, or cementing the ceramic facet or crowns prepared in the laboratory on the fractured part of the restoration without removing the restoration from the mouth [13]. The low cost and ease of application of composites used in the direct repair method are beneficial. However, disadvantages such as low durability, lack of resistance to abrasion, and poor aesthetics limit the use of composites for intraoral repair [14]. Despite its disadvantages, such as high cost and inferior technical precision compared to composite repair materials, the durability of ceramics, their lower cost compared to the replacement of a prosthesis, and their high aesthetic properties are among the advantages of the indirect method [13].

The aim of this study is to compare the bond strength of porcelain laminate veneers to be used in the indirect repair method, which is a more aesthetic and durable option, and composite resins used in the direct repair method applied in the clinical routine for repairing zirconia and veneered zirconia FDPs.

## 2. Material and Methods

### 2.1. Specimen Preparation

**Figure 1:**
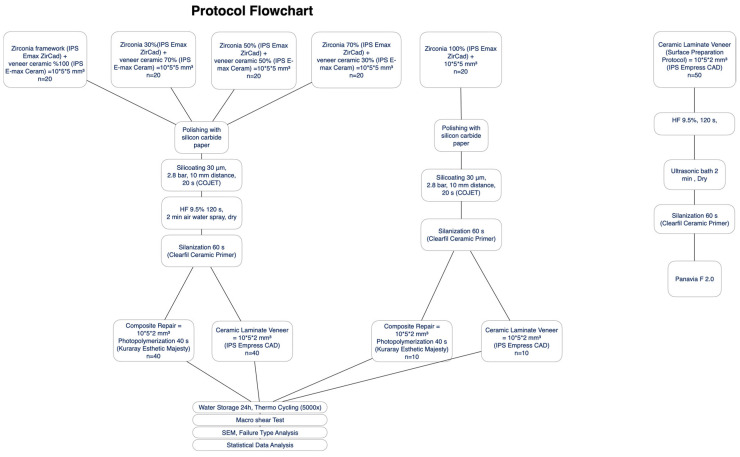
Experimental flowchart presenting the distribution of the tested groups according to testing method, substrate, and procedural sequences of the experiment.

The experimental flowchart explaining the distribution of the specimen groups in the study regarding substrate type, testing method, and experimental procedure sequences is shown in Figure 1.

Material brands, manufacturers, types, and chemical compositions of all products used in the study are presented in Table 1.

One hundred zirconia specimens from IPS e.max ZirCad blocks (Ivoclar Vivadent, Schaan, Liechtenstein), that would form the substructures to be used in the study, were made in a special laboratory in accordance with the manufacturer’s instructions, in the desired dimensions and shapes, using a CAD/CAM system (InLab MC X5, Sirona Dental Systems, GmbH, Bensheim, Germany). To evaluate the two repair methods in laboratory conditions, substructures in different configurations were prepared to simulate different fracture types.

From the specimens, a total of 10 subgroups were formed by dividing each main group into 2 subgroups. Half of the groups were repaired directly with composite resins, and the other half were indirectly repaired with porcelain laminate veneers (Table 2).

**Table 2:** Specimen Groups.

	Laminate Veneer Repair	Composite Resin Repair
**Gr A = 100% Zr (n:20)**	Gr A1	Gr A2
**Gr B = 70% Zr (n:20)**	Gr B1	Gr B2
** Gr C = 50% Zr (n:20) **	Gr C1	Gr C2
** Gr D = 30% Zr (n:20) **	Gr D1	Gr D2
**Gr E = 0% Zr (n:20) **	Gr E1	Gr E2

**Figure 2:**
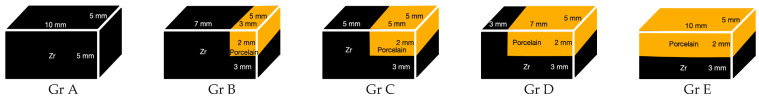
Representative images of all groups.

Representative images of the samples are given below in Figure 2. Black areas refer to zirconia, greyareas refer to porcelain.

#### 2.1.1. Zirconia Preparation

Drawings with the *.stl extension for the Zr substructures were transferred to the AutoCAD program, and they were scraped from the CAD/CAM system (InLab MC X5, Sirona Dental Systems, GmbH, Bensheim, Germany) using IPS e.max ZirCad (Ivoclar Vivadent, Schaan, Liechtenstein) blocks in desired sizes and shapes. The sintering process was applied at 1500 °C for 16 h.

#### 2.1.2. Application of Suprastructure Ceramics

Suprastructure ceramics were not applied to Group A, that was made out of these substructures. A silicone mold was prepared for the areas left for porcelain addition to Groups B, C, and D, and 2 mm thick porcelain was applied to Group E (e.max Ceram porcelain powder, IPS e.max Ceram Build Up liquid, Ivoclar Vivadent, Schaan, Liechtenstein) and fired in a porcelain furnace at 755 °C.

After the porcelains were applied to the Zr substructure groups, a plexiglass cube was formed, and silicone molds were prepared to place them in cold acrylic using a standardized procedure. Afterwards, the surfaces of the samples to be repaired were placed in the middle of the silicone base, and cold acrylic was poured on them.

#### 2.1.3. Surface Treatments of Specimens

After all specimens were placed in acrylic molds, they were numbered separately, and the surface treatments were applied. To ensure that the surfaces of the samples were smooth and even and to ensure standardization, 600-, 800- and 1200-grit silicon carbide abrasives (English Abrasives, English Abrasives Ltd., London, England) were used in this order with a polishing machine (MiniTech 233, PRESI, Eybens, France) with water at 300 rpm.

The specimens were air-abraded (Cojet, 3M ESPE, St. Paul, MN, USA) with Al_2_O_3_ coated with silica for 20 s from a distance of 10 mm at a pressure of 2.8 bar using an intraoral air-borne particle abrasion machine (Microetcher, Danville, VA, USA). Afterwards, they were washed and rinsed.

After air-abrasion, the specimens in Groups B, C, D, and E were treated with 9.6% HF acid (Ultradent, South Jordan, UT, USA). Each specimen was acidified for 120 s, washed with water for 2 min, and dried.

#### 2.1.4. Preparation of Laminate Veneers

Drawings were prepared in AutoCAD for the indirect facet application method. Fifty laminate samples to be used in the study were made of IPS EmpressCad blocks (Ivoclar Vivadent, Schaan, Liechtenstein) in a special laboratory in accordance with the manufacturer’s instructions, in the desired dimensions and shapes with a CAD/CAM system (InLab MC XL, Sirona Dental Systems, GmbH, Bensheim, Germany).

The surfaces of the laminate veneers to be used for cementation were etched with 9.6% HF acid (Ultradent, South Jordan, UT, USA) for 120 s. After etching, the laminates were washed with distilled water for 120 s in an ultrasonic cleaner and air-dried.

### 2.2. Cementation of Laminate Veneers for Indirect Technique

Since the specimens in Groups A1, B1, C1, D1, and E1 would be repaired by indirect facet application, the laminates were cemented to these number 1 subgroups. Half of the samples in Groups A, B, C, D, and E were randomly selected for inclusion in the first subgroups. These groups, whose surface preparations were completed, were first treated with silane for 60 s (Clearfil Ceramic Primer and Clearfil SE BOND primer were mixed in a one-to-one ratio according to the manufacturer’s instructions (Kuraray Medical Inc., Tokyo, Japan). The dual cure cement of choice (Panavia F2.0, Kuraray Medical Inc., Japan) was then applied according to the manufacturer’s instructions. It was applied by mixing according to the specifications and cemented under constant pressure by placing a weight of 750 g on the laminates. The residual cement was cleaned, and the specimen was polymerized for 20 s on each surface.

### 2.3. Applying Composite Resins for Direct Technique

The composite material was applied to the specimens in Groups A2, B2, C2, D2, and E2. The groups were treated first with silane for 60 s. Clearfil Ceramic Primer and Clearfil SE BOND primer, mixed one to one according to the manufacturer’s instructions (Kuraray Medical Inc., Japan), were applied to the remaining specimens of Groups A, B, C, D, and E. After application of Clearfil Ceramic Primer and Clearfil SE BOND primer mixture, Clearfil SE Bond (Kuraray Medical Inc., Japan) was applied and polymerized for 40 s. A 2 mm thick composite (Kuraray Esthetic Majesty, Kuraray Medical Inc., Japan) was applied to each sample surface with reference to the 2 mm high silicone matrix that was formed. After the matrix was removed, each sample was polymerized for another 20 s on each surface.

### 2.4. Aging

The specimens were subjected to thermal cycling to imitate intraoral temperature changes and obtain results closer to in vivo conditions. After keeping all specimens in distilled water for 24 h, 5000 cycles were applied made in a 5–55 °C water bath in accordance with ISO standards, keeping the specimens in each bath for 30 s and allowing a 5 s transition period.

### 2.5. Adhesion Tests and Mode of Failure Analysis

**Figure 3:**
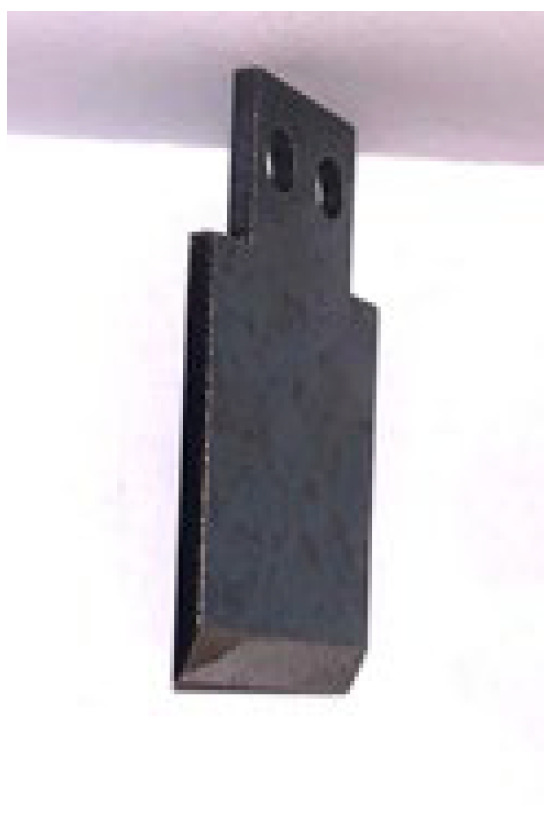
Specially made tip for Universal Testing Machine.

**Figure 4:**
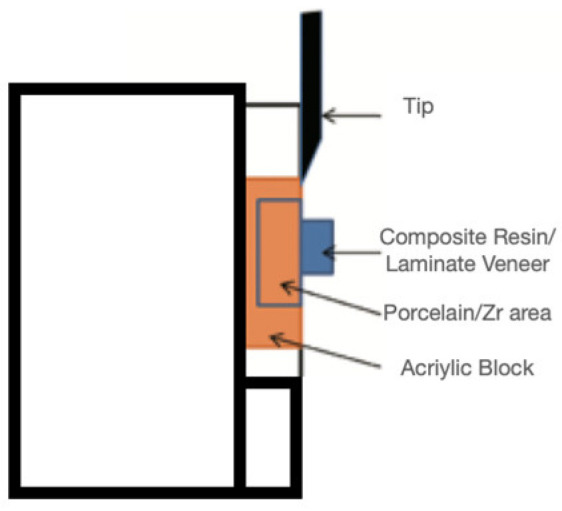
Universal Test Machine using simulation with the tip.

After the thermal cycling process, the specimens were placed in the universal testing device (Instron, Schimadzu Autograph Ag-50 Kng and Ag-500) for the measurement of their shear bond strength values. After a specially made tip (Figure 3), thinned on one side, was placed in the device, the part of this tip that would touch the bonding interface was adjusted, and the speed of the tip was set at 0.5 mm/min (Figure 4). The values measured during separation were recorded in Newtons and calculated in MPa by dividing the result by the surface area of the laminate and composite materials.

After the shear test, the surfaces of the specimens were examined with a microscope (Axio Zoom V616, Zeiss, Oberkochen, Germany), and each group was separated according to their modes of failure.

### 2.6. Statistical Analysis

The data were analyzed using IBM SPSS V23 (IBM Corp. Released 2015. IBM SPSS Statistics for Windows, Version 23.0. Armonk, NY: IBM Corp.). Conformity to normal distribution was evaluated with the Shapiro–Wilk test. An independent-samples *t*-test was used to compare the normally distributed data. The normally distributed data are presented as mean ± standard deviation. The level of statistical significance was taken as *p* < 0.050.

## 3. Results

### 3.1. Shear Bond Strength Results

Shear bond strength test results of each sample were collected by Universal Test Machine. Then average values of each group were calculated statistically according the observed results (Table 3).

As seen in the results above, the shear bond strength values of the subgroups repaired using laminate veneers were higher than the values of those repaired using composite resins, and these differences were statistically significant in all groups except for Group C.

### 3.2. Stereomicroscopic Analysis Results

**Figure 5:**
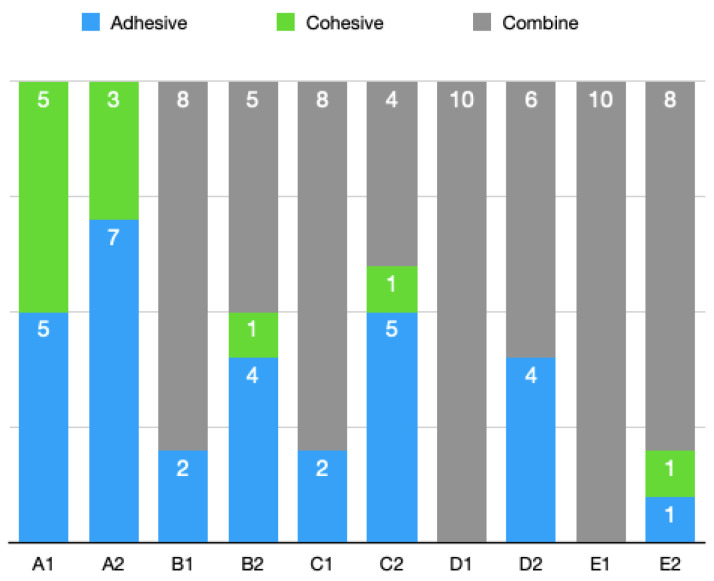
Fracture types (adhesive/cohesive/combine) comparison in all groups.

**Figure 6:**
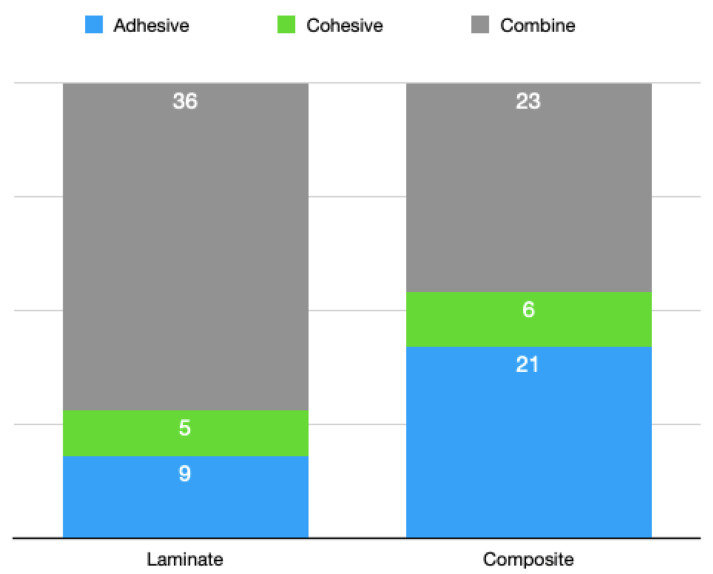
Fracture types (adhesive/cohesive/combine) comparisons of laminate veneer and composite resin groups.

Fracture types (modes of failure) were divided into the adhesive, cohesive, and combine categories by imaging. As in the tables below, all composite groups had more specimens with the adhesive fracture type than the laminate groups did (Figure 5). Both of the groups had approximately similar specimens with the cohesive fracture type. Laminate veneer groups had more specimens with the combine fracture type than the composite groups did (Figure 6).

## 4. Discussion

Today, the use of all-ceramic prosthetics has increased compared to conventional metal-supported prostheses. This is because, in addition to the increased aesthetic expectations of patients, the biocompatibility of all-ceramic restorations is also higher. Zirconium oxides, which are among all-ceramic systems, have high durability, can be used in posterior teeth, and are indicated for multi-member bridge construction. In cases where aesthetics is important, they are often preferred because of their light transmittance and the absence of reflection from the gingiva in, as opposed to the case in conventional metal prostheses [15]. The increase in the use of zirconium oxide has attracted more studies. It has been observed that the failure rate due to fatigue in zirconia-based restorations is higher than that in metal-ceramic restorations [16]. One of the most common failures in zirconia-based restorations is the separation of the superstructure porcelain from the substructure in layers (delamination) or by breaking (chipping) [7]. Chipping and delamination are considered to be related to the tensile forces at the zirconium–porcelain interface. The reasons for this stress are the difference between the thermal expansion coefficients of the substructure and superstructure porcelains, the inability of the superstructure porcelain to wet the substructure sufficiently, the shrinkage that occurs during firing, the heat-induced phase transformation of zirconia crystals, low thermal conductivity, low surface roughness, and others [3]. Apart from this, trauma and occlusal conflicts can also cause fractures or fragmentations [7]. The need for repair on this material has gained importance due to the increase in the frequency of construction of zirconia-based restorations and the higher fracture rate in long-term follow-up studies compared to those on metal-based restorations [17].

In the clinic, fractures of porcelain restoration with zirconia substructures can be seen as fractures that occur only on the porcelain surface, where a part of the substructure is exposed with the fracture in the porcelain, a large part of the porcelain is broken, and the entire substructure is exposed [10]. For this reason, to imitate cases of fracture in the mouth in this study, the samples to be tested were prepared not only on porcelain surfaces but also at different proportions of porcelain and zirconium. All clinically encountered situations were evaluated separately in terms of their bond resistance values.

When a restoration is broken, a complete repair is the ideal approach. However, the difficulty of removing the restoration, the potential of damage to the supporting teeth during removal, increased costs, and loss of time may be listed as some disadvantages for the patient and the physician. Removing the restoration from the mouth and repairing it in the laboratory is also a treatment option. In this option, too, the abutment teeth are damaged, and distortions may occur when re-firing the restoration. The restoration can maintain its functions in the mouth after a fracture, and if there is no other reason for its reproduction, the option of repairing it in the mouth provides convenience for the patient and the physician [18].

This study was aimed at observing the benefits from using intraoral repair systems. Intraoral porcelain repairs can be made directly or indirectly. Composite resins are used in direct techniques, and adhesive materials are used in indirect techniques [19]. To ensure the bonding of the repair material to the substructure, preparations for the ceramic surface are required in clinical applications. In our study, the same surface preparations were applied to porcelain samples with zirconium substructures, and the bond strength values of the direct and indirect repair processes were investigated. Along with technological developments, the use of new methods and new materials should be tested without losing their currency. This can be achieved with faster, easier, and more economical in vitro studies [20]. Based on this, our study was conducted under in vitro conditions. In vitro tests should be supported by clinical studies for many reasons such as material properties, patient-related variables, dynamic occlusal loads, and fatigue phenomena. However, patient follow-up is required in clinical studies, and this takes time. Additionally, different stresses in the oral environment, such as heat, blood, gingival crevicular fluid, bruxism, daily functions, thermal stresses, and malocclusion, affect bonding and do not allow the identification of the factor causing the failure [21].

Specimen sizes were determined as 10 × 5 × 5, based on the study conducted by Sang J. Lee et al. in 2014 [22,23]. Since the specimens specified in ISO 11,405: 1994(E) must imitate the intraoral conditions, an average incisor size and a laminate veneer are assumed to be used [24].

While superstructure porcelain is being applied on zirconium substructures, the characteristics of the ceramic that is used and the technical sensitivity of its production affect the fracture state of the porcelain [25]. The layering technique is a precision technique and can be affected by variables such as the technician’s experience, number of firings, and cooling time [26]. In our study, surface treatments were applied after the application of the superstructure porcelain, and the same technician performed these procedures under the same conditions.

Surface preparation processes are performed to increase the bonding between the repair material and the ceramic structure [19]. These surface treatments provide micromechanical and chemical bonding between the ceramic and the repair material [27]. With surface treatments, microroughness is provided mechanically, and surface tension is reduced by increasing the surface roughness, which allows mechanical/chemical bonding. Chemically, with the physical change obtained by dissolving the glass matrix, the resin is bonded to the porous surface [28].

Various surface treatments such as roughening with diamond burs, sandblasting with Al_2_O_3_, hydrofluoric acid/phosphoric acid treatment, laser application, and airflow can be used on zirconium and porcelain surfaces. Sandblasting with silica-bonded Al_2_O_3_ powder can be used to provide chemical adhesion as well as mechanical retention. In addition to these, silane and adhesive primers are also used to increase bonding strength [29].

One of the surface preparation processes, roughening with a diamond bur, creates micromechanical retention. This process is low-cost and easily implemented [30]. The adhesive agent leaks into the recesses that appear after this process. It is used in the diamond bur repair process to remove the unsupported porcelain on the surface [18]. After this process, sharp irregularities occur on the surface. At the same time, microcracks that are formed after the process can cause fractures [31]. Furthermore, high stress, decrease in bending strength, and phase transformation can be seen on the zirconia surface in the roughening processes that are carried out with a diamond bur [32]. It has been stated that surface abrasion with a diamond bur should generally be preferred in intraoral procedures where sandblasting is not possible [33]. In our study, to avoid the disadvantages of using a diamond bur on zirconia and porcelain surfaces, it was not included in the surface treatments. One of the surface treatments applied in intraoral repair is sandblasting with Al_2_O_3_ particles. Air-abrasion is one of the most common methods used to increase the surface roughness of zirconia [34]. Air-abrasion with Al_2_O_3_ creates micromechanical retention on the porcelain surface and physically changes the surface [35], increasing the surface area and allowing the surface to be wetted by the resin [36]. When Al_2_O_3_ particles are applied to the zirconia surface, transformation from the tetragonal phase to the monoclinic phase can cause erosive wear and fractures. In their study in 2005, Guazzato et al. observed that air-abrasion increased the flexural strength of zirconia despite the damage it may cause on the surface [37]. Air-abrasion has been applied tribochemically with the CoJet system (3M ESPE, Seefeld, Germany), and it has been seen that silane treatment increases the bonding to zirconium oxide ceramics compared to Al_2_O_3_ air-abrasion [36]. The main effect of tribochemistry is that the material displays chemical and physicochemical changes during the application of mechanical energy. This technique was developed with the thought that the bond strength of metal and oxide ceramics that do not contain silanol groups will increase if they are silanized [38]. Atsu et al.’s study in 2006 supported this view [39]. The shear strength between zirconia and composite resin was examined, and the highest bond strength was in the group where air-abrasion was performed with the CoJet system and silane application [40]. In our study, the connection was standardized at the highest level by tribochemical coating on the zirconia surface specimens with the CoJet system.

Acid etching, another form of surface treatment, increases the surface area and energy of the ceramic, and the bonding rate of the resin to the ceramic increases with the surface energy [41]. Previous studies have shown that acid etching increases resin bonding in glass ceramics. Surface preparation with 5–10% HF in porcelain repair is one of the preferred methods to increase the bond between a restoration and a resin [42]. HF acid is effective in silica-containing ceramics, but highly crystalline materials such as zirconium oxide and aluminum oxide are resistant to such treatments because they lack a glassy phase and are devoid of silica [43]. Studies have shown that HF acid application does not increase the strength of the bond between zirconia and resin [44].

In this study, each specimen was etched for 120 s. This treatment on the zirconia surface was made to clean the material, while it was made on the porcelain surface to strengthen the bond. Although the use of HF acid in the mouth is controversial due to its harmful effects on tissues, there are no reports of complications related to HF acid in the dentistry literature [45]. HF acid treatment may protect patients from possible unwanted restoration outcomes. At the same time, resin-containing materials will be prevented from absorbing water and changing their physical properties. After HF acid was applied to the samples, the samples were cleaned with an ultrasonic cleaner and freed from residues.

It has been reported that in order to strengthen the bond between the zirconia surface and the repair resin, the surface should be wetted with a phosphate monomer (MDP monomer) following surface treatments, so that a chemical bond can be provided, in addition to the micromechanical bond [46]. It has been shown that a phosphate monomer can form chemical bonds with metal oxides. In this case, a strong bond can be achieved by forming a chemical bond of zirconia with the phosphate monomer [46].

In their study in 2005, where the effects of the CoJet system were examined, Bottino et al. compared air-abrasion alone, silica coating, and silane applications containing a silane bonding agent and reported that the tensile strength between the resin cement containing a phosphate monomer and the zirconium ceramic was higher in the silica-coated specimens [47]. In some studies, it was stated that the application of silane after the application of the CoJet system increased the bond strength of silica-containing, glass-infiltered alumina and zirconium oxide ceramics [47]. In our study, it was aimed to establish a durable bond by applying the silane binding agent to the specimen surfaces after the CoJet application.

Composite resins are often used for porcelains that fail in intraoral repair systems, and there are many systems developed for this purpose [23]. However, this type of repair is seen as temporary because a decrease in bond strength has been observed in the long term. In vitro studies have also shown that bond strength decreases after aging processes [30]. Additionally, the prognosis is controversial in direct intraoral repair methods carried out with composites, due to the wear of the composite over time and the lack of color stability with ceramics [14]. In our study, the groups in which the specimens underwent composite-based repair were considered the control groups, since this repair method is frequently performed in the clinic, but due to the disadvantages of this method, the option of repair with ceramic laminates was also examined.

In the intraoral direct repair method with composite resins, the type of composite that is used affects the bond [48]. While microfilled composites can be used in repair systems at the level of enamel porcelain, condensable hybrid-derived composites are used in posterior region fractures that will be exposed to chewing pressure [1]. Microfilled composites are produced for use in the anterior region and are preferred in anterior restorations due to their high light transmittance and high suitability for polishing [49,50]. In our study, Clearfil Majesty Esthetic (Kuraray), a microfilled composite resin, was preferred because of its superior aesthetic properties and studies showing its adequate bonding properties [49]. Composites wear out over time and can also undergo physical and chemical changes [51]. Color harmony deteriorates with the surface change of the composite resin. Additionally, the consumption of products containing coloring agents such as tea, coffee, and cigarettes, improper surface polishing, and poor oral hygiene cause discoloration in restorations [52]. With the purpose of obtaining a smooth finishing surface in composite resin restorations, studies on polishing have been and continue to be conducted frequently [53]. Pratten et al. reported that smoother surfaces were obtained with tires in their study in 1988 [54]. According to the study conducted by Horton et al. in 1977, suitable polishing materials are discs and polishing tapes [55]. The achievement of a smooth surface varies according to the brand of the composite, the type of filler, particle size, the amount of filler, the type of resin, and the characteristics of the polishing tool [56]. In 2021, Szalewski et al. observed that influence of beverages had impacts on the mechanical properties of resin composites [57]. In addition, another study of Szalewski et al. has shown that type of polymerisation affects the mechanical properties of resin composites [58]. Disadvantages of the finishing process, the wear of composites over time and changes in surface properties by beverage impacts, affect the coloration of the surface in intraoral repairs with composite resins, and were some of the reasons why we compared composites and porcelain laminates in our study. The fact that the bond strength values were found to be significantly higher in four of the groups that were repaired with laminate veneers and that the polishing and finishing processes of porcelain veneers were made by firing in the laboratory, and the polished surfaces were not deteriorated, showed that the laminate repair method was superior to the composite repair method in our study.

As another intraoral repair system, the repair method with laminate veneers, also known as indirect facet application, has a higher success rate than composite resins in which the direct repair method is used, especially in cases where the ceramic contains large fractures [59]. The fact that the aesthetic properties of full ceramics are more favorable than composite resins, that they show color stability and are not corroded, and that their bonding to the substructure in repair is sufficient in previous studies, has led to the thought that the application of porcelain repair with laminate veneers may increase in clinical use [13] Porcelain laminates are aesthetically superior materials with long-term and predictable results with appropriate patient selection and experienced technicians [60]. The introduction of new materials for CAD/CAM systems and developments in adhesion techniques allow laminate veneers to be applied with CAD/CAM systems in a single session, resulting in more conservative treatments that are made possible [61]. The clinical success rate of laminate veneers produced with CAD/CAM systems was shown to be 99% in a 5-year period [60]. It has been reported that laminate veneers made using Empress Cad and Emax Cad did not show any difference in terms of marginal fit, periodontal response, or coloration [62].

Surface treatment of porcelain laminate veneers with HF acid on the surface to be bonded before cementation increases the bond strength by providing micromechanical adhesion [63]. It has been scientifically proven that both micromechanical bonding and chemical bonding can be achieved adequately with the application of silane binder to laminate veneers [64]. In this study, silane was applied after HF acid was applied to the inner surfaces of the laminates. This way, it was aimed to increase the bond strength.

Many cements that can be used for bonding laminates in the indirect repair method have been developed. In a previous study, it was shown that the surface treatments applied to zirconia affected bond strength more than the cement did [65]. Another study compared the bond strength of Panavia F 2.0 to that of Variolink on the zirconia surface and revealed a higher bond strength in the Panavia F 2.0 [66]. In this study, in light of this information, Panavia F2.0 cement with MDP content was preferred for indirect facet application.

Composite resin cements shrink during polymerization, which causes stresses in the thin cement layer that provides the adhesive bond [67]. These stresses may exceed bond strengths and put the life of the restoration at risk [68]. Furthermore, additional stresses in the cement, such as stresses due to biting forces on the cemented restoration, will increase the likelihood of bond failure. Therefore, the design of the cement layer is important. Nevertheless, more research is needed to assess the stressors that arise in the clinical environment [18]. The thickness of the cement plays an important role in these stresses in cementation processes. Finger pressure or weight can be used for the standardization of cement thickness in in vitro studies. A previous study showed that the finger pressures applied by dentists varied between 12 and 67 N and showed a statistically significant difference compared to the finger pressure applied during cementation [69]. In another study, the effects of forces that were applied during cementation on the cementation process were examined, and it was revealed that there was no statistically significant difference among 10 g, 50 g, 100 g, 500 g, and 750 g forces [68]. It has been stated that in the oral environment, the dimensions of cements change due to water absorption, so the use of thick cement will affect the duration of the restoration process [68]. In this study, the cementation of the samples was achieved using a 750 g weight, and standardization was achieved in terms of cement thickness.

In a previous study, differences were detected in the measurements made immediately after polymerization and 24 h after the adhesion test was applied. Measurements made immediately after cementation yielded lower values and were shown to be due to incomplete polymerization [70,71].

In our study, after the samples were cemented, they were kept in distilled water for 24 h to avoid losing their moisture and to complete the polymerization process. It was reported that mixed-type fractures were more common in groups with high shear bond strength values, and adhesive fractures became more common as bond strength values decreased [72,73]. In another study, it was shown that mixed-type and cohesive fractures were preferred more than adhesive fractures, and adhesive fractures were associated with low bond strength [39]. In line with studies in the literature that have reported different distributions of adhesive, cohesive, mixed-type fractures, we believe that there is no single generally accepted view. Therefore, it is necessary to increase the number of studies on this topic.

It was stated that intraoral porcelain repair materials applied on a metal alloy exhibited much less bond strength than those applied on porcelain surfaces [74]. In studies involving different porcelain repair systems, results ranging from 6 to 29.9 MPa have been reported in bond strength tests [28]. The bond strength values in another study were reported in the range of 1–17 MPa [75]. In this study, values in the range of 2.4 to 12.1 MPa were obtained in the bond strength tests in two different repair methods. The results of our study were similar to those reported in other studies. Differences in roughening methods, differences in sample sizes, surface areas, and types of repair materials used may be the reason for differences in results. In some studies, it has been reported that the strength of the bonds between composites and oxide or metal surfaces is less than that between composites and porcelain surfaces [9,11]. It was stated that the amount of porcelain remaining on the fractured surface is important in bonding. The fracture surfaces formed after the test applied in a previous study were generally observed at the metal–composite interface [11]. Similarly, in our study, adhesive type fractures in the samples generally occurred at the zirconium–composite or zirconium–resin cement interface. It was observed that as the percentage of porcelain on the surface increased, the rate of mixed-type fractures in the repair systems increased. In patients undergoing intraoral porcelain repair, regardless of direct or indirect repair, if there is a parafunction, a stabilization splint should be prepared to prevent the recurrence of the fracture after the repair. Thus, the repair area can be protected from destructive occlusal forces.

## 5. Conclusions

In this study, composite resin and porcelain laminates and surfaces containing porcelain and zirconia at different proportions were repaired. The bond strength values of direct and indirect porcelain repair systems in zirconia-supported ceramics were compared, and the following results were obtained:

- When entire zirconia substructure was exposed, the success of the porcelain repair made with laminate veneers was higher than that of the repair with the composite.
- In the group where most of the zirconia substructure was exposed, the success of the porcelain repair made with laminate veneers was higher than that of the repair with the composite.
- In cases where the zirconia and porcelain surfaces were at equal ratios, there was no statistically significant difference between the repair made with the composite or the repair made with the laminate veneers, but the laminate veneers had numerically higher bond strength values.
- In the group where a small part of the zirconia substructure was exposed, the success of the porcelain repair made with the laminate veneers was higher than that of the repair with the composite.
- When the zirconia substructure was not exposed, and the surface was completely made of porcelain, the bonding success of the porcelain repair made with the laminate veneers was higher than that made with the composite.
- The subgroups repaired with the composite showed more adhesive fractures than the subgroups repaired with the laminate veneers. This distribution was directly proportional to the bond strength values.

## Figures and Tables

**Table 1:** Product names, manufacturers, and the respective chemical compositions of the study material.

Product Name	Manufacturer	Chemical Composition
IPS e.max CAD ZirCad	Ivoclar Vivadent AG, Schaan, Liechtenstein	Zirconium dioxide (87–95), Yttrium oxide (4–6), Hafnium oxide (1–5), Aluminum oxide (0–1)
IPS e.max Ceram	Ivoclar Vivadent AG, Schaan, Liechtenstein	Silicon dioxide (60–65), Aluminium Oxide (9–11), Potassium oxide (7–8), Sodium oxide (7–8), Zinc Peroxide (2–3), Oxocalcium, Phosphorus pentoxide and Fluorine (2.5–7.5)
IPS Empress CAD	Ivoclar Vivadent AG, Schaan, Liechtenstein	Silicon dioxide (60–65), Aluminium Oxide (16–20), Potassium oxide (10–14), Sodium oxide (3.5–6.5), other oxides (0.5–7.0), pigments (0.2–1.0)
Panavia F 2.0 Paste A	Kuraray Medical Inc., Japan	10-Methacryloyloxydecyl dihydrogen phosphate, hydrophobic aromatic dimethacrylate, hydrophobic aliphatic dimethacrylate, hydrophilic aliphatic dimethacrylate, silanized silica filler silanized colloidal silica, dl-camphorquinone, catalysts
Panavia F 2.0 Paste B	Kuraray Medical Inc., Japan	Hydrophobic aromatic dimethacrylate, hydrophobic aliphatic dimethacrylate, hydrophilic aliphatic dimethacrylate, silanized barium glass filler, catalysts, accelerators, pigments
Clearfil Ceramic Primer	Kuraray Medical Inc., Japan	3-trimethoxysilylpropyl methacrylate (5), ethanol (80), other ingredients: 10-methacryloyloxydecyl dihydrogen phosphate
Clearfil SE Bond Primer	Kuraray Medical Inc., Japan	2-hydroxyethyl methacrylate (10–30), other ingredients: 10-methacryloyloxydecyl dihydrogen phosphate Hydrophilic aliphatic dimethacrylate dl-camphorquinone, accelerators, water, dyes
Clearfil SE Bond, Bond	Kuraray Medical Inc., Japan	bisphenol A diglycidylmethacrylate (25–45), 22-hydroxyethyl methacrylate (20–40), other ingredients: 10-methacryloyloxydecyl dihydrogen phosphate, hydrophobic aliphatic dimethacrylate, colloidal silica, dl-camphorquinone initiators, accelerators
Hydrofluoric Acid	Ultradent, USA	9% hydrofluoric acid
Clearfil Esthtetic Majesty Composite	Kuraray Medical Inc., Japan	bisphenol A diglycidylmethacrylate (Bis-GMA), other ingredients: silanized barium glass filler, pre-polymerized organic filler, hydrophobic aromatic dimethacrylate, hydrophobic aliphatic dimethacrylate, dl-camphorquinone, accelerators, initiators, pigments
Cojet Sand	3M Espe, USA	Silica-coated sand
Silica Carbide Abrasives	English Abrasives	Cubiron and aluminum mineral

**Table 3:** Shear bond strength comparisons of laminate veneers and composite resins in each groups.

	Mean ± S. Deviation (MPa)	Median (Min–Max) (MPa)	Test Statistics	* p *
A1	12.1 ± 4.4	11.6 (3.8–18.8)	t = 4.147	0.001
A2	6.1 ± 1.4	6 (3.8–7.9)		
B1	7.1 ± 3.1	7.4 (2.2–10.8)	t = 2.707	0.020
B2	4.3 ± 1	4.4 (2.3–5.5)		
C1	6.3 ± 3.6	5.4 (2.3–13.6)	t = 1.775	0.103
C2	4.2 ± 1.3	4.1 (2–6.1)		
D1	7.1 ± 2.2	6.5 (4.9–10.8)	t = 5.394	<0.001
D2	3.1 ± 0.8	3 (1.8–4.3)		
E1	6.7 ± 3.1	6.2 (3.6–12.9)	t = 4.237	0.002
E2	2.4 ± 0.8	2.6 (1.1–3.6)		

## Data Availability

The data presented in this study are available on request from the corresponding author. The data are not publicly available due to privacy.
